# Treatment of Painful Palmoplantar Keratoderma Related to Pachyonychia Congenita Using EGFR Inhibitors

**DOI:** 10.3390/biomedicines10040841

**Published:** 2022-04-03

**Authors:** Céline Greco, Anne-Charlotte Ponsen, Stéphanie Leclerc-Mercier, Joël Schlatter, Salvatore Cisternino, Claude Boucheix, Christine Bodemer

**Affiliations:** 1 Department of Pain and Palliative Care, Hôpital Necker-Enfants Malades, Assistance Publique Hôpitaux de Paris (AP-HP), 75015 Paris, France; 2 IMAGINE Institute, Inserm U1163, Université de Paris, 75015 Paris, France; anne-charlotte.ponsen@inserm.fr; 3 Department of Pathology, Reference Center for Genodermatoses (MAGEC), Hôpital Necker-Enfants Malades, Assistance Publique Hôpitaux de Paris (AP-HP), 75015 Paris, France; stephanie.leclerc@aphp.fr; 4 Service Pharmacie, Hôpital Paul Doumer, Assistance Publique Hôpitaux de Paris (AP-HP), 60332 Liancourt, France; joel.schlatter@aphp.fr; 5 Service Pharmacie, Hôpital Necker-Enfants Malades, Assistance Publique Hôpitaux de Paris (AP-HP), 75015 Paris, France; salvatore.cisternino@aphp.fr; 6 Faculté de Pharmacie, Optimisation, UMRS-1144, Université de Paris, INSERM, 75006 Paris, France; 7 Inserm UMRS-MD-1197, Université Paris-Saclay, 94800 Villejuif, France; claude.boucheix@inserm.fr; 8 Department of Dermatology, Reference Center for Genodermatoses (MAGEC), Hôpital Necker-Enfants Malades, Assistance Publique Hôpitaux de Paris (AP-HP), 75015 Paris, France

**Keywords:** pachyonychia congenita, palmoplantar keratoderma, pain, EGFR, tyrosine kinase inhibitors

## Abstract

Pachyonychia congenita (PC) is a genodermatosis associated with severe painful palmoplantar keratoderma (PPK) and thickened dystrophic nails caused by autosomal dominant-negative mutations in five genes encoding keratins 6A-B-C, 16, and 17. The mechanical, surgical, or medical options for painful PC are inefficient. Given ErbB/Her family members’ role in epidermal homeostasis, this study sought to investigate the possibility of treating PC patients with PPK by blocking signaling either with EGFR (Her1) inhibitor erlotinib or lapatinib, a dual EGFR(Her1)/Her2. After 1 month of therapy with oral erlotinib treatment at 75 mg/day, the pain disappeared for patient #1, with partially reduced hyperkeratosis, while increasing the dose to 100 mg/day resulted in painful skin fissures. Therapy replacement with erlotinib cream at 0.2% was inconclusive, and substitution with oral lapatinib at alternating doses of 500 and 750 mg/day achieved a good compromise between pain reduction, symptom improvements, and side effects. Patient #2′s treatment with erlotinib cream failed to display significant improvements. Oral erlotinib started at 75 mg/day then reduced to 25 mg/day because of the formation of an acneiform rash. Treatment considerably improved the patient’s condition, with an almost complete disappearance of pain. Oral Her1 or 1/2 inhibitors reduced pain, improved two PC patients’ quality of life, and offered promising therapeutic perspectives.

## 1. Introduction

Pachyonychia congenita (PC) is a rare genodermatosis characterized by keratinization disorders resulting from autosomal dominant-negative mutations in five genes encoding keratins (K6a, K6b, K6c, K16, or K17) specific for keratinocyte differentiation, each defining one PC subtype. All individuals with PC present palmoplantar keratoderma (PPK) restricted to pressure points in palmar or plantar epidermis, leading to dramatic, painful thickening and hyperkeratosis. Regarding this main symptom, PC is associated with thickened dystrophic nails, oral leukokeratosis, follicular hyperkeratosis, and multiple cysts. Elevated mechanical stress in combination with the physiological presence of K6, K16, and K17 in the palmoplantar epidermis is a part of the explanation for the site predilection. However, the pathophysiology of PC-associated PKK is only partially understood. Additionally, an inflammatory cascade has been described in a PC-PKK in a mouse model [1,2]. Although some of these symptoms, such as thickened nails, tend to occur in the first year of life, disease diagnosis is often delayed due to the large variability in phenotypes [3,4,5]. To date, numerous treatments to manage the painful PKK of PC have been tested, including retinoids, surgical and mechanical interventions, orthotics, keratolytics, opioids, antidepressants, antiepileptics, and even botulinum toxin [6]. Accelerated research on PC has resulted in developing experimental targeted therapeutic strategies, such as small interfering RNAs targeting mutant keratin alleles and reducing their expression, or mTOR inhibition by rapamycin/sirolimus suppressing K6a expression [7]. These treatments have not yet reached routine clinical use [5,6]. Two cases reporting successful oral rosuvastatin treatment in patients displaying a KRT6A mutation were published [8,9]. However, larger trials are still pending due to negative results observed with the statin approach [10]. PC is a very disabling condition because of the intensity of the pain. Therefore, new efficient therapeutic approaches are required and decidedly expected by the patients concerned.

Epidermal hyperplasia has drawn research attention to ErbB receptors, particularly for EGFR signaling, which plays a critical role in skin homeostasis by regulating the proliferation of basal epidermal cells and their differentiation at the supra-basal level [11,12]. Previously, we described a PPK remission with erlotinib, an EGFR inhibitor, in three patients with Olmsted syndrome (OS), a severe genodermatosis linked to activating TRPV3 mutations that usually develops in childhood [13]. This approach was based on the observation that TRPV3 induces EGFR transactivation through ADAM 17 activation and cleavage of the EGFR ligand precursor, namely, TGFα [14]. Pain reduced within days, with near-complete remission of the patients’ keratoderma at three months. Similar clinical results were reported by other investigators [15,16].

Considering the role of EGFR in normal skin, as well as its functional exacerbation in painful PPK of Olmsted disease, we wondered whether the intense and painful PPK observed in PC may similarly involve increased EGFR signaling despite the absence of an identified link between keratin mutations and EGFR activation. With this hypothesis in mind, we administered systemic or topical erlotinib treatments to two PC patients while investigating whether this approach could improve hyperkeratosis and associated symptoms, as observed in OS. In one patient, erlotinib was relayed by lapatinib, a dual EGFR(ErbB1)/Her2 inhibitor. Her2(ErbB2) is an orphan EGFR family receptor which dimerizes with its partners, particularly EGFR, for signaling [12].

## 2. Methods

In a prospective intervention, which was not registered as a clinical trial, we treated 2 patients suffering from PC with EGFR inhibitors. Patient #1 received oral and topical EGFR inhibitors (erlotinib, Roche, Grenzach-Wyhlen, Germany), which were then replaced by the oral EGFR/Her2 inhibitor (lapatinib, Novartis Europharm Limited, Dublin, Ireland). Patient #2 received topical then oral erlotinib. For topical treatment, a 0.2% erlotinib cream (oil-in-water emulsion) was applied twice a day to each area of hyperkeratosis (1 g per foot). Plasma dosages were performed. Generally, targeted residual plasma concentrations of erlotinib in cancer patients are 1200–2000 ng/mL (daily oral doses of 150 mg for adults), whereas the range of steady-state concentrations achieved with 1250 mg oral lapatinib (single dose in fasting patients) are 910 ± 520 ng/mL [17,18]. Since many factors affect the absorption of these drugs, particularly food ingestion, patients were asked to take a single daily dose one hour before meals. Both patients were informed of the molecules’ nature and potential undesirable effects prior to signing their written informed consent.

Immunohistochemical staining was performed with antiphospho-ERK antibody (phospho-p44/42 MAPK XP Rabbit mAb, Cell Signaling, dilution 1/400) on a Bond III Leica automate (Leica Microsystems SA, NanterreFrance). Formalin-fixed, paraffin-embedded slides were pretreated using epitope retrieval solution 1, with citrate-based pH of 6 for 20 min followed by antibody incubation for one hour at room temperature. Revelation was performed using Bond Polymer detection kit with DAB.

Pain severity was assessed using the numeric rate scale pain score (NRS, scale 0–10) ranging from ‘no pain’ to ‘worst imaginable pain’. Neuropathic pain was evaluated more specifically with the Neuropathic Pain Symptom Inventory (NPSI), a self-questionnaire specifically designed to evaluate the different symptoms of neuropathic pain [19]. This questionnaire is comprised of ten descriptors scored from 0 to 10. Combining these descriptors into five factors allows a five-subscale representation, where each factor, scored from 0 to 10, represents a different dimension of neuropathic pain: superficial spontaneous pain (burning), deep spontaneous pain (pressing), paroxysmal pain, evoked pain, and paresthesia/dysesthesia. A total intensity score, from 0 to 100, is the sum of the scores of the 10 descriptors. The five subscores correspond to the mean scores of the descriptors belonging to each of the five factors [19]. Quality of life before and after treatment was assessed by using the Dermatology Life Quality Index (DLQI), a validated, self-administered, and user-friendly ten-question questionnaire designed to measure the health-related quality of life of adult patients suffering from a skin disease [20]. Each question is scored from 0 to 3, giving a possible total score range from 0 (meaning no impact of skin disease on quality of life) to 30 (meaning maximum impact on quality of life).

## 3. Results

**Figure 1:**
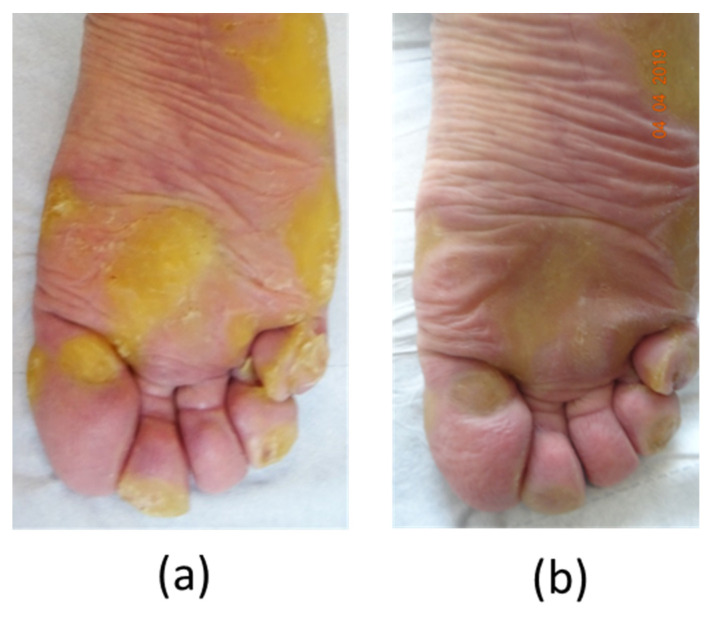
Evolution of plantar keratoderma in patient #1. (**a**) Skin lesions in patient #1 at day 0 before erlotinib treatment. (**b**) Hyperkeratosis reduction after 2 months of treatment.

**Patient#1**, a 47-year-old man, displayed significant focal PPK (Figure 1a). A causative mutation was identified in KRT16, which carried one heterozygous c.374A > G substitution (NM_005557.3) in exon 1, leading to p.Asn125Ser.

**Figure 2:**
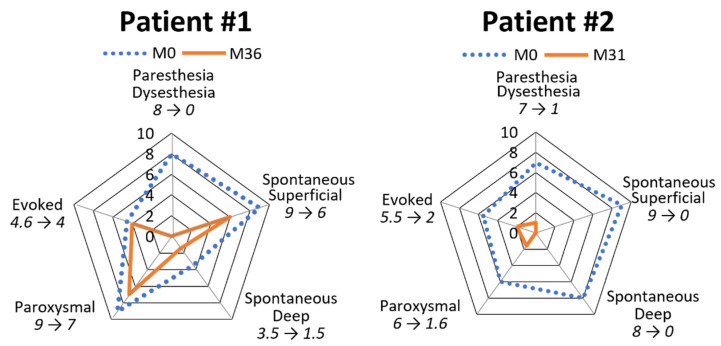
Graphical representation of Neuropathic Pain Symptom Inventory. The evolution of the five factors is shown from the beginning of treatment to present for the two patients, and the variation in score is indicated below each factor (M = month).

**Figure 3:**
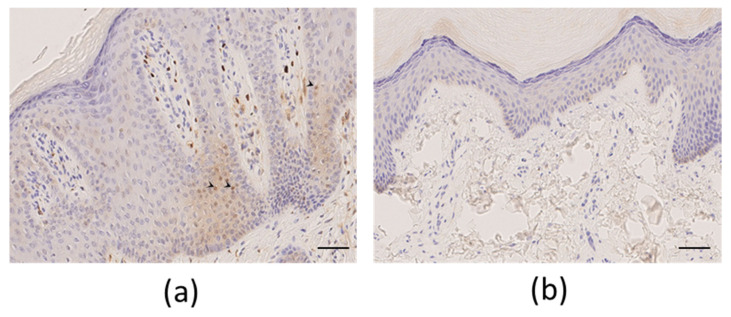
Phospho-extracellular-signal-regulated K-kinase (ERK) labeling—immunohistochemistry with anti-phospho-ERK antibodies. (**a**) Nuclear staining (arrowheads) of numerous keratinocytes in the supra-basal layer of hyperplastic epidermis (original magnification 40×) before erlotinib treatment in patient #1. (**b**) Absence of nuclear staining in normal thick skin biopsy. Scale bar 50 µm.

Palmoplantar keratoderma developed progressively since childhood. There were no other associated symptoms (mostly no dystrophic nails, oral leukokeratosis, follicular hyperkeratosis, and multiple cysts). His mother had similar symptoms, but no genetic test could be performed. The patient’s pain was resistant to strong opioids, antidepressants, and antiepileptics. Topical keratolytics and oral sirolimus were both poorly effective. Retinoids (Acitretin) significantly reduced the plantar hyperkeratosis, but these agents were associated with remarkably poor tolerance, even at low-dose levels (<0.5 mg/kg), resulting in undesirable effects such as superficial acral erosions, painful cracks (fingertips and toes), and marked cheilitis occurring within 1 month of treatment. The disease’s burden was major, with the NRS pain score between 7/10 and 10/10, and the patient experienced difficulty walking and working, with a DLQI score of 17/30. The NPSI was scored at 64/100 with a predominance of spontaneous superficial pain (scored at 9/10), paroxysmal pain (9/10), and paresthesia/dysesthesia (8/10), while spontaneous deep pain (3.5/10) or evoked pain (4.6/10) were less pronounced (Figure 2). A plantar skin biopsy showed phospho-ERK expression by epidermal cells, which was not seen in control thick skin, indicating activation of the extracellular-signal-regulated kinase (ERK)/mitogen-activated protein kinase (MAPK) pathway (Figure 3). Erlotinib treatment was initiated on February 8, 2019, at an initial posology of 75 mg/day, according to its usage in other diseases. After one month of therapy, we observed a spectacular reduction in pain (NRS pain score 0/10), resulting in the discontinuation of all analgesics. We noticed a significant decrease in hyperkeratosis, with a markedly decreased inflammatory border (Figure 1B), enabling the patient to walk longer distances. Moreover, regular mechanical debridement with skin cut-out was no longer necessary. Erlotinib was tolerated well despite acneiform lesions on the face and thoracic area, which progressively regressed with a classical topical treatment for acne. The residual blood concentrations varied between 237 and 684 ng/mL.

In March 2019, oral erlotinib doses were increased to 100 mg/day. Nevertheless, the complete disappearance of hyperkeratosis could not be achieved, as previously observed in OS, whereas the onset of acral painful cracks required a reduction in erlotinib doses to 75 mg/day.

In October 2019, at month 8, the decision was made to investigate whether applying topical erlotinib could induce similar results, thereby allowing for oral treatment discontinuation. As a result, the patient discontinued oral 75 mg/day erlotinib intake and started to apply 0.2% erlotinib cream (oil-in-water emulsion) twice a day to each hyperkeratosis area (1 g per foot). Despite a slower regrowth of the hyperkeratotic plaques, a major recurrence of pain was observed, probably due to a too-low topical concentration of erlotinib, forcing the patient to resume oral treatment at 75 mg/day. Despite a satisfactory effect on pain and hyperkeratosis, undesirable cutaneous effects manifested again. Due to the insufficient efficacy of topical treatment and undesirable effects of the oral formulation, we then switched from erlotinib to lapatinib. Oral lapatinib was initiated on 1 August 2020, at 250 mg/day, which was then progressively increased to 1000 mg/day over one month. At the beginning of September 2020, we observed a remarkable pain reduction (NRS 1/10) and a decrease in hyperkeratotic plaques, whereas cracks in the fingertips and epistaxis reappeared. The blood concentration of lapatinib was measured at 1692 ng/mL. Oral lapatinib was reduced to 750 mg in November 2020, until December 2020, when dosing was reduced to alternate 500 and 750 mg doses that attained equilibrium between pain reduction in the absence of analgesics and undesirable effects. This dosage did not change for a year. In January 2022, the patient had no more difficulty working and much less difficulty walking with the possibility of family hikes. He scored the DLQI at 9/30. His NPSI changed considerably, with a total score of 35/100 (the subscale representing paresthesia/dysesthesia scored at 0/10, spontaneous superficial pain at 6/10, paroxysmal pain at 7/10, spontaneous deep pain at 1.5/10, and evoked pain at 4/10) (Figure 2).

**Figure 4:**
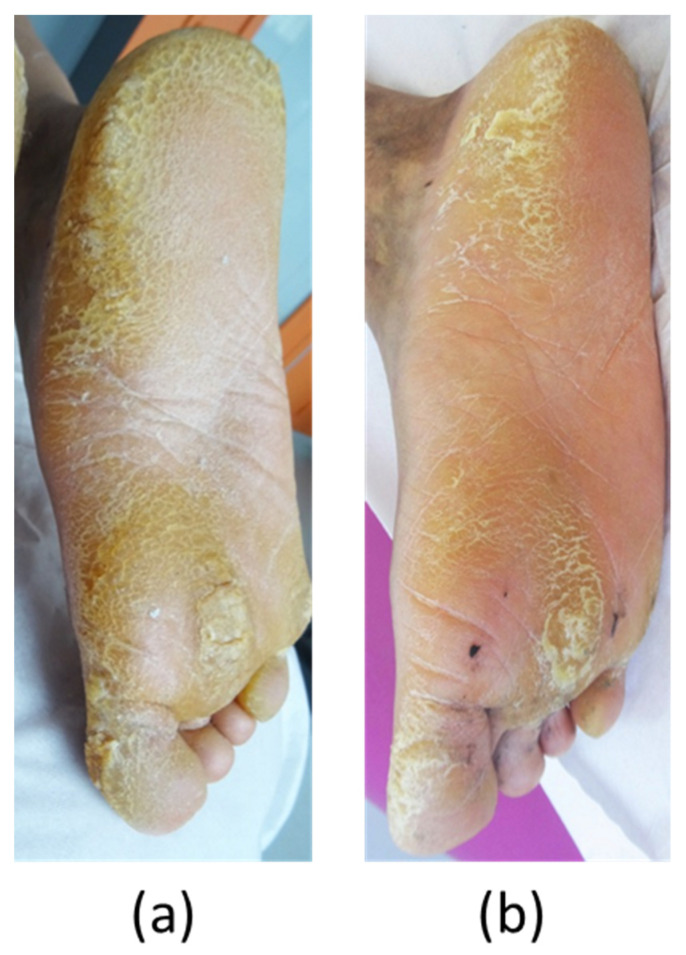
Evolution of plantar keratoderma in patient #2. (**a**) Skin lesions in patient #2 on day 0 before oral erlotinib initiation (3 February 2020). (**b**) Hyperkeratosis reduction after 11 months of treatment (3 January 2021).

**Patient#2**, a 39-year-old man, exhibited significant focal PPK (Figure 4a). A causative mutation in KRT6A was identified, which carried one heterozygous c.516_518 deletion (NM_005554) in exon 1, leading to p.Asn172del without familial history. Painful palmoplantar keratoderma developed since infancy with acute episodes of pain, leading to crawling in childhood. The PPK was associated with dystrophic nails, oral leukokeratosis, follicular hyperkeratosis, but no cysts. In adulthood, the patient suffered from permanent and severe pain with NRS pain scored at 7/10 and huge difficulty walking, with the DLQI scored at 25/30. The NPSI was measured at 71/100 with a predominance of spontaneous superficial (scored at 9/10) and deep (8/10) pain, paresthesia/dysesthesia (7/10), and paroxysmal pain (6/10), while evoked pain (5.5/10) was less pronounced (Figure 2). His pain was resistant to strong opioids, antidepressants, and antiepileptics. Topical keratolytics and oral retinoids (up to 0.75 mg/Kg) were ineffective. Topical 0.2% erlotinib therapy was initiated on 10 July 2019, consisting of 1 g of erlotinib cream applied twice a day on the right foot. Pain and hyperkeratosis were compared with those of the left foot, which was left untreated. At one month, the patient observed a diminution in pain on his right foot (NRS 4/10), compared with the left foot (NRS 7/10). Hyperkeratosis thickening was improved, and mechanical debridement by the patient was eased. Nevertheless, debridement was still required two to three times per month. We did not observe a remarkable improvement in the nail’s dystrophy and follicular keratosis; oral leukoplakia remained stable.

Residual blood concentrations of erlotinib following topical administration were undetectable. Owing to the topical application’s failure to induce further improvement, we initiated oral erlotinib therapy at 75 mg/day on 3 February 2020. After one month of therapy, the erlotinib blood concentration was 1233 ng/mL. We observed complete pain relief (NRS 0/10), whereas mechanical debridement involving skin cut-out was less frequently required (once/month). However, in April of the same year, an acneiform rash imposed treatment discontinuation, which was restarted at 25 mg/day in July 2020 at the patient’s request because of severe pain recurrence (NRS 6/10) and a reduced walking perimeter (500 m). After 3 months of therapy, the erlotinib blood concentration was 235 ng/mL, the pain was below 1/10, and the walking perimeter was augmented up to 6 km, without any undesirable effects noticed by the user. In January (Figure 4b) and July 2021, the patient’s condition was further improved, along with reduced hyperkeratosis, absent pain, and a 10 km walking perimeter. In January 2022, pain was no longer present, and the patient had only taken one pill of tramadol in 6 months with no debridement for 6 months. He scored the DLQI at 13/30. The NPSI was measured at 10/100. Spontaneous superficial and deep pain disappeared (both scored at 0/10), paresthesia/dysesthesia were almost non-existent (scored at 1/10), along with paroxysmal pain (1.6/10) and evoked pain (2/10) (Figure 2).

## 4. Discussion

These two observations of patients suffering from very PPK in PC with different keratin mutations indicate that oral EGFR inhibitors may improve their condition. A larger clinical trial will be necessary to confirm these promising preliminary results. A question of concern is the high cost of tyrosine kinase inhibitors therapy, but erlotinib lost patent protection in 2019, and generic prices considerably lower its cost. The same should soon apply for lapatinib.

Pain symptoms described by our patients are suggestive of neuropathic pain, as mentioned earlier in a series of PC patients [21,22]. Although pain was greatly improved, including symptoms related to neuropathic pain, partial disappearance of hyperkeratosis plaques could only be achieved in parallel to the occurrence of skin fissures in one patient and intolerance to erlotinib doses in the second. Of note is that the first patient displayed a particular sensitivity to low-acitretin dosing, with the same secondary acral cracks. Topical erlotinib was tested as a potential therapeutic option to avoid undesirable systemic effects. Although an improvement was observed, this approach was poorly effective and would presumably have required drug concentrations above 0.2%, the latter being a technical challenge because of the drug’s low solubility. However, significant improvement with topical erlotinib treatment was recently reported in two K16 PC patients, with an effect more pronounced in one of them [23]. Considering disorders with altered keratinocytes’ mechanical functions related to keratin mutations, it is interesting to emphasize that hyperkeratosis could be a secondary protective mechanism of skin fragility [24]. Reducing PPK too intensely may unmask skin fragility in PC, contrary to what is observed in OS. Therefore, for PC patients, it is necessary to find the right balance between EGFR inhibition and skin-protective reactions, as well as between sufficient pain relief and triggered skin fragility symptoms.

More generally, PPK syndromes are genodermatoses linked to mutations in different genes expressed in keratinocytes. The discovery that TRPV3 signaling requires EGFR transactivation [11] has led to a successful attempt at treating Olmsted syndrome linked to TRPV3 activating mutations with erlotinib [13]. Independently of the genetic cause, PPK is usually associated with the triad of hyperkeratosis, pain, and inflammation symptoms. If the development of hyperkeratosis is biologically understandable given the role of EGFR in the proliferation and differentiation of keratinocytes [12], the mechanism of inflammation and pain remains poorly explored. In OS, where EGFR transactivation appears to be the main signaling pathway leading to skin manifestations of the disease, erlotinib treatment results in the rapid disappearance of pain preceding the regression of inflammation and near-total disappearance of hyperkeratosis. In PC, where the primary genetic mutations affect keratins involved in epidermal repair, it is difficult to significantly reduce hyperkeratosis without unmasking symptoms of skin fragility. On the other hand, a clear effect on pain is obtained early in treatment and is sufficient to restore a good quality of life.

These observations raise questions about the link between ErbB receptors, mainly EGFR, and the emergence of pain and inflammation. The implication of ErbB receptors in pathological pain, particularly EGFR, has been extensively reviewed by Borges et al. [25], as well as some aspects of the relation with inflammation. The ability of EGFR inhibitors to exert analgesic effects on neuropathic pain has been tested in animal models of nociceptive responses and is supported by empiric clinical findings in cancer and non-cancer patients [26,27]. ErbB receptors are expressed on sensory neurons (afferent nerve fibers) and non-neuronal cells of the nervous system (spinal microglia, satellite cells of rat DRG) and may be activated by endogenous or exogenous ligands. However, even if TRPV3 is suggested to be expressed on DRG cells, transactivation of EGFR, as observed in keratinocytes, has not been reported in these cells.

The question raised by these observations is how keratinocytes may mediate a pain signal to nociceptor pathways. The secretion of pain mediators by keratinocytes has been reported in TRPV3 activation independently of its mechanism activation (ligand or activating mutation). Various nociceptor activators are released, including PGE2, ATP, NGF, NO, IL-4, IL-13, IL-17 [28], IL18 [22], SP, and CGRP [29]. Additionally, some proinflammatory cytokines released by keratinocytes, such as IL1α, IL6, IL8, and TNFα, involving the keratinocyte NF-κB pathway, may mobilize inflammatory cells [30]. Inflammatory cells could then alter sensory fibers, provoking neuropathic pain [29]. The keratinocytes’ intracellular signaling pathways leading to pain or inflammatory soluble mediators’ release are only partially identified, but the clinical effect of erlotinib strongly suggests that EGFR signaling is involved in their secretion, at least in OS and PC. Inflammatory reactions could also be induced by the structural alteration of keratinocytes due to keratin mutations that render the cells more fragile and may lead to the release of damaged associated molecular patterns (DAMP) [2]. A direct effect of EGFR ligands released by keratinocytes should also be investigated, as well as the expression and function of ErbB receptors on nociceptive nerve fibers.

## 5. Conclusions

The report of these two cases of PC treated with erlotinib opens very promising therapeutic perspectives for these rare yet very disabling keratin disorders and offers new areas of research in the field of cutaneous pain.

## Data Availability

Not applicable.
